# Study on Gas Chromatographic Fingerprint of Essential Oil from *Stellera chamaejasme* Flowers and Its Repellent Activities against Three Stored Product Insects

**DOI:** 10.3390/molecules26216438

**Published:** 2021-10-25

**Authors:** Yuli Sang, Jingyu Liu, Lei Shi, Xiulan Wang, Yueqiang Xin, Yanjun Hao, Li Bai

**Affiliations:** 1 College of Pharmacy, Liaoning University, Shenyang 110036, China; sangyuli@lnu.edu.cn (Y.S.); jingyu163ying@163.com (J.L.); shileifts@163.com (L.S.); xinyq0802@163.com (Y.X.); 2 Inner Mongolia Institute of Mongolian Medicine Engineering Technology, Inner Mongolia University for Nationalities, No. 536 West Huolinhe Street, Tongliao 028000, China; tlmdwxl@163.com; 3 Liaoning Academy of Traditional Chinese Medicine, Liaoning University of Traditional Chinese Medicine, No. 79 Middle Chongshan Road, Shenyang 110032, China; 4 Market Supervision Service Center, Qianshan District, Anshan 114041, China

**Keywords:** *Stellera chamaejasme* flowers, essential oil, chromatographic fingerprint, chemical composition, repellent activity, stored product insects

## Abstract

The objective of this study was to establish the chromatographic fingerprints of the essential oil (EO) from *Stellera chamaejasme* flowers collected from various natural sites by gas chromatography (GC) combined with chemometric methods. The EO was obtained by hydrodistillation, and its chemical composition was analyzed by gas chromatography−mass spectrometry (GC−MS). Most components were identified as ketones and the relatively high-content components were fitone (38.973%), *n*-hentriacontane (5.807%), myristic acid (4.944%) and phytol (3.988%). In addition, the repellent activities of the EO from *S. chamaejasme* flowers and its four main chemical compounds were evaluated against three stored product pests (*Tribolium castaneum*, *Lasioderma serricorne*, *Liposcelis bostrychophila*) for the first time. In this work, the EO and the four chemical compounds showed a repellent effect against three storage pests after 2 and 4 h exposure. The experimental method and repellent activity of *S. chamaejasme* flower EO could provide a basis for the development of botanical pesticide and the utilization of the rich plant resources of *S. chamaejasme* in the future.

## 1. Introduction

*Stellera chamaejasme* (L.) is a perennial herb of the genus *Stellera* in the *Thymelaeaceae* family. Its root is commonly used for removing phlegm, relieving pain, killing pests and for external use in the treatment of scabies in traditional Chinese medicine (TCM) [1]. *S. chamaejasme* mainly grows on dry sunny hillsides, lawns, or riversides with an altitude range from 2600 to 4200 m above sea level, mean annual temperature of about 0 °C in the north and southwest of China, as well as in Siberian Russia. Previous studies have found that the root parts of *S. chamaejasme* contain flavonoids, lignans, coumarins, terpenes and other chemicals [2,3], among which coumarins are its characteristic chemical components such as daphnetin, umbelliferone, edgeworthin, etc. However, there is relatively little research into the chemical composition of the flower part of *S. chamaejasme*. Because *S. chamaejasme* has strong survivability and expansion capacity on degraded grasslands, aggravating grassland desertification, it is generally considered as a grassland desertification indicator plant [4,5,6]. In addition, studies showed the whole plant of *S. chamaejasme* was toxic, as a result, poultry and livestock were often found to be poisoned in pastures by misusing *S. chamaejasme*, which bring economic loss and a safety hazard to the urban resident and herdsmen [7,8].

Red flour beetle *Tribolium castaneum* (Herbst) (Coleoptera: Tenebrionidae), cigarette beetle *Lasioderma serricorne* (Fabricius) (Coleoptera: Anobiidae) and booklouse *Liposcelis bostrychophila* (Badonnel) (Psocoptera: Liposcelididae) are worldwide and destructive pests of stored products, especially found in tropical and subtropical areas [9,10,11]. Due to their strong adaption, rapid breeding ability and worldwide distribution, the above three insects have caused serious problems including agricultural economic loss, food contamination and health risks to consumers, etc. [12,13]. Therefore, pesticides such as synthetic insecticides have been widely used to control these stored product pests, in the meantime, overuse of synthetic chemical insecticides leads to environmental pollution, unexpected toxicity on the nontarget biosome and endangerment of human health [14,15]. Consequently, it is urgent to study new green and safe pesticides; natural plants have gained widespread interest in pest control on account of the insecticidal potential and easy degradation of their secondary metabolites [16,17,18]. Nowadays, the development of botanical insecticides has been recognized as an efficient and environmentally acceptable alternative strategy for pest management.

Research has suggested that many plants possess insecticidal activities. For example, *Cinnamomum* plants showed remarkable bioactivities against pests, and the EO of *C. zeylanicum* was highly poisonous to some mosquito vectors and flies [19]. The EO from roots and leaves of *Asarum heterotropoides* also displayed toxicity and repellency against the cigarette beetle and booklouse [20]. Additionally, some species of the *Rhododendron* genus, such as *R. anthopogonoides*, *R. thymifolium*, *R. capitatum* were all reported to have insecticidal properties, and the EO extracted from the species of *Rhododendron* was utilized against stored product pests [21]. According to the earliest Chinese pharmaceutical classics, the *Shennong* herbal scripture, the whole plant of *S. chamaejasme* is poisonous, and its pollen is a highly toxic part, which suggests the EO of *S. chamaejasme* flowers could have the potential activity of a pesticide and the development of an insecticide may also provide an approach for the reasonable utilization of *S. chamaejasme* plant resources [22,23,24].

As is known, TCM has been widely used in the treatment of human diseases for a long time and has played an important role in clinical practice due to its therapeutic action, low toxicity and few side effects up to now. One characteristic of TCM products which is different from synthetic drugs is their complex and unstable chemical constituents, because the chemical composition of TCM products is always affected by many factors including the producing areas, harvest time, growth environment and field management [25], resulting in a batch-to-batch variation to different degrees. In addition, the active components and the action mechanism of some TCM drugs are still unclear. Therefore, it is of great significance to establish the scientific quality control methods of TCM drugs based on their global chemical features, which could give support for identification, quality assessment and provide guidance for clinical medication. The combination of a chromatographic fingerprint and chemometrics analysis could be an effective way for the quality evaluation of TCM products, especially for judging the authenticity of TCM products by similarity evaluation [26]. By analyzing the relative retention time and peak area or intensity ratio of the common peaks, the chemical profile and fingerprint of TCM drugs are established integrally and comprehensively. According to the chromatographic fingerprints, the similarities and differences of various TCM products can be intuitively compared. On the other hand, the chemometric method has become one of the popular modern analysis methods, because it can provide more information on the samples based on the chromatographic fingerprint. The correlation coefficient in chemometrics is the common index for evaluating the similarity of different samples, which can represent the difference and similarity between a set of chromatographic fingerprints and help to classify the quality traits by comparing it with the average reference values. Consequently, the systematic clustering analysis method has been widely used in the data analysis of chromatographic fingerprints, detecting the chromatographic fingerprint information more accurately and comprehensively [27].

In this study, GC−MS was used to determine the chemical constituents of the EO from *S. chamaejasme* flowers for the first time, and the gas chromatographic fingerprint combined with the chemometric method was successfully developed and applied to assess the quality consistency of seven batches of EOs from *S. chamaejasme* flowers. By using the Chinese medicine chromatographic fingerprint similarity evaluation system (China National Pharmacopoeia Commission), the common characteristic peaks of *S. chamaejasme* flowers EOs were confirmed and the similarities of *S. chamaejasme* flowers from different areas were analyzed. In addition, the chromatographic fingerprint data of *S. chamaejasme* flowers EO were systematically clustered to evaluate the consistency and differences of the samples. In the past, research on the *S. chamaejasme* plant mainly focused on the chemical composition and pharmacological activities of the medical root part, while studies on the above-ground parts of this herb, particularly the research into the flowers of *S. chamaejasme* were rarely reported [28,29]. In the light of the toxicity and the reported insecticidal effects of the root [30,31], our research team extracted the EOs of *S. chamaejasme* flowers from various natural sites and evaluated their repellent activities against three worldwide pests (*T. castaneum*, *L. serricorne*, and *L. bostrychophila*). Meanwhile, the repellent activities of the four main chemical compounds of the EO against these three pests were also measured. By analyzing the chromatographic fingerprints and repellent activities of the EO from *S. chamaejasme* flowers, this study showed the similarities of seven batches of *S. chamaejasme* and could also offer enlightenment for the development of botanical pesticides.

## 2. Results and Discussion

### 2.1. Chemical Components of Essential Oil

The chemical composition of the EO from *S. chamaejasme* flowers was analyzed by GC−MS. Twenty chromatographic peaks were totally isolated from the EO of *S. chamaejasme* flowers. Sixteen chemical components were identified, accounting for 96.667% of the total peak area, after searching through the NIST 05 Standard Spectral Library data system and checking the mass spectrum fragmentation law. The analysis results are shown in Table 1. Among the identified components in the EO of *S. chamaejasme* flowers, the content of ketone compounds was higher in proportion, accounting for 50.894% of the identified ingredients, and the acids, alcohols and alkanes accounted for 10.811%, 9.938% and 5.870% respectively.

### 2.2. Establishment of Chromatographic Fingerprints and Selection of Reference Peaks

**Figure 1:**
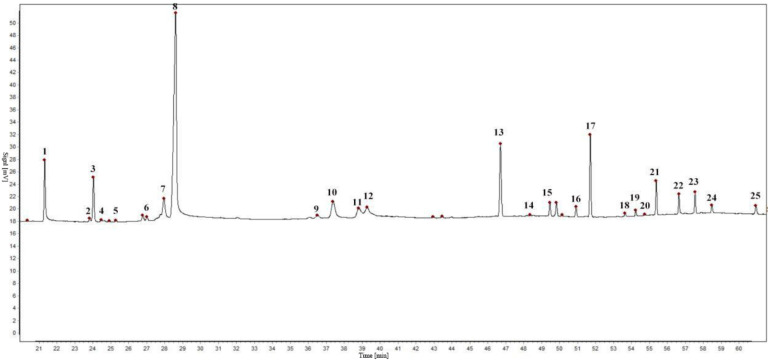
Gas chromatogram of *S. chamaejasme* flowers.

The EOs from seven batches by the establishment of chromatographic fingerprints and selection of reference peaks of *S. chamaejasme* flowers in different natural sites were determined by GC. The experimental results showed that 25 characteristic peaks of the EOs were confirmed as common chromatographic peaks shown in Figure 1, accounting for more than 96% of the total peak area. Among the common chromatographic peaks, three peaks respectively named characteristic peak 1, 8, and 13, labeled in Figure 1, were identified as myristic acid, fitone and *n-*hentriacontane by GC−MS.

It is necessary to select an appropriate peak as the internal reference peak in the complex chromatographic peaks of the samples, for digitizing the retention values of the chromatographic peak into relative retention values [32,33]. In the GC fingerprint of the EO from *S. chamaejasme* flowers, the myristic acid peak was chosen as the internal reference peak.

### 2.3. Calculation of Chromatographic Peak Retention Values and Similarity Analysis of Chromatographic Fingerprints

**Figure 2:**
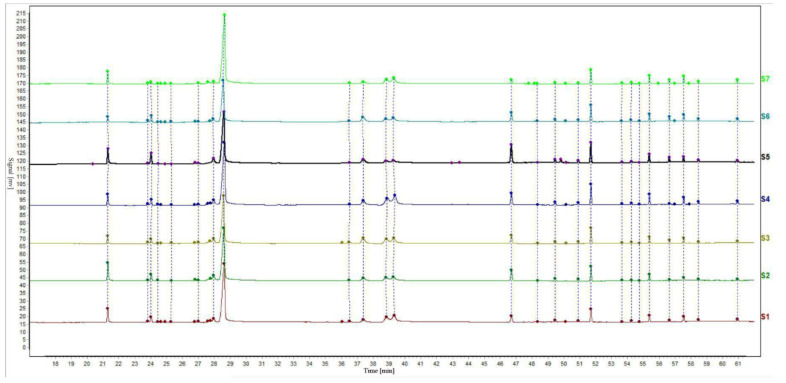
Gas phase fingerprint of essential oil from *S. chamaejasme* flowers.

The relative retention time and relative peak area of the chromatographic peaks were calculated according to the selected internal reference peak. The relative retention time and relative peak area of the common fingerprint peaks of the EOs from seven batches of *S. chamaejasme* flowers are presented in Table 2 and Table 3. The relative retention time of the common peak is the ratio of the absolute retention time of the characteristic peak to that of the reference peak, and the relative peak area of the common peak is found in the same way. In addition, the gas-phase fingerprints of seven batches of *S. chamaejasme* flower EOs from different natural sites in Inner Mongolia were analyzed with the ‘Chinese Medicine Chromatographic Fingerprint Similarity Evaluation System’ (Chinese Pharmacopoeia Committee) for similarity evaluation [34,35]. The chromatogram is shown in Figure 2. The internal reference peak of myristic acid was peak No. 1 and the median method was used to calculate the similarity. Sample No. 1 was taken as the reference template. According to the peak matching results, the calculated results of all similarities between the complete fingerprints of the tested samples and those of the control template are shown as Table 4. The results showed that the similarity value of the seven samples was more than 0.9. Therefore, the fingerprint maps of the seven batches of EOs were basically consistent, which indicated that the qualities of the seven batches of *S. chamaejasme* flowers from various natural sites of Inner Mongolia autonomous region in China were basically the same. The results can provide a reference for the qualitative identification of *S. chamaejasme* herbs and clinical application of *S. chamaejasme* flowers.

The percentage values of the peak area of the common and noncommon peaks of the seven tested samples were shown in Table 5. The results indicated that the common peak area of the seven samples accounted for more than 95% of the total peak area, and the noncommon peak area account for less than 5%. Hence, the above experimental results were officially valid and also meet the requirements of fingerprinting [36,37].

### 2.4. Multivariate Data Processing and Analysis

**Figure 3:**
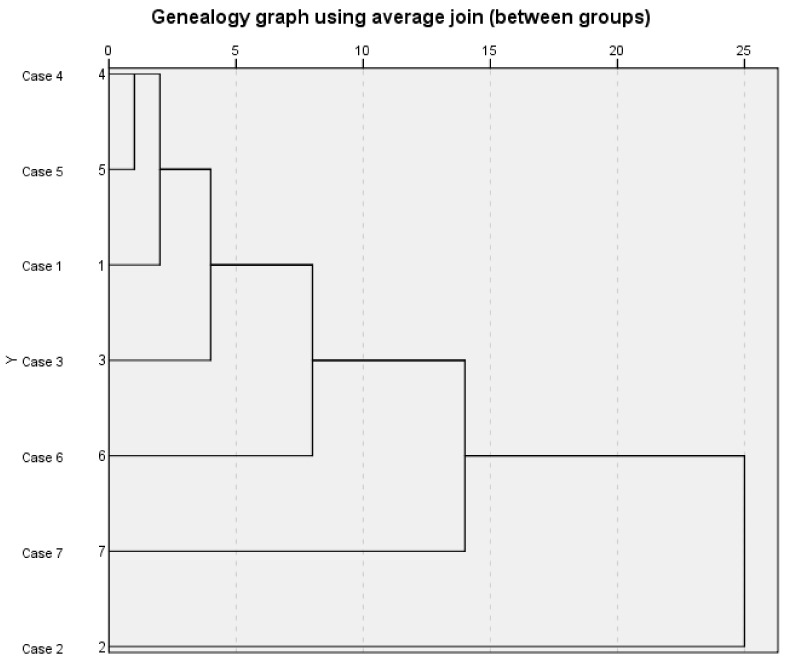
Systematic cluster analysis pedigree of 7 batches of *S. chamaejasme* flowers samples.

Systematic clustering analysis was carried out on seven samples of *S. chamaejasme* flowers. After standardized processing of the peak areas of 25 common peaks in the fingerprint, SPSS 26.0 software was used to perform systematic clustering analysis with the average connection of groups and the squared Euclidean distance as metric standard [38,39,40]. The results are shown in Figure 3. The abscissa of the system cluster analysis pedigree diagram represents the relative distance between groups, and the ordinate represents the sample numbers. The pedigree diagram of system cluster analysis can visually show the process of the samples merging gradually [41,42]. Among them, when the distance between groups was 5, the seven batches of samples could be divided into four types, sample numbers 2, 6, and 7 belonged to their respective type, while Nos. 1, 3, 4 and 5 were one class which indicated that the quality of *S. chamaejasme* flowers in these four natural sites was relatively consistent.

### 2.5. Repellent Activity

The chemical composition of EO from *S. chamaejasme* flowers was identified and analyzed by GC−MS, the content of compound fitone (38.978%) was the highest, followed by myristic acid (4.944%), methyl palmitate (4.854%), and phytol (3.988%). The results of the tested samples are presented in Table 6. Additionally, in this study, the insecticidal activity of the EO of *S. chamaejasme* flowers and its main chemical components were evaluated by using the target insects of the *T. castaneum*, *L. serricorne*, and *L. bostrychophila*.

**Figure 4:**
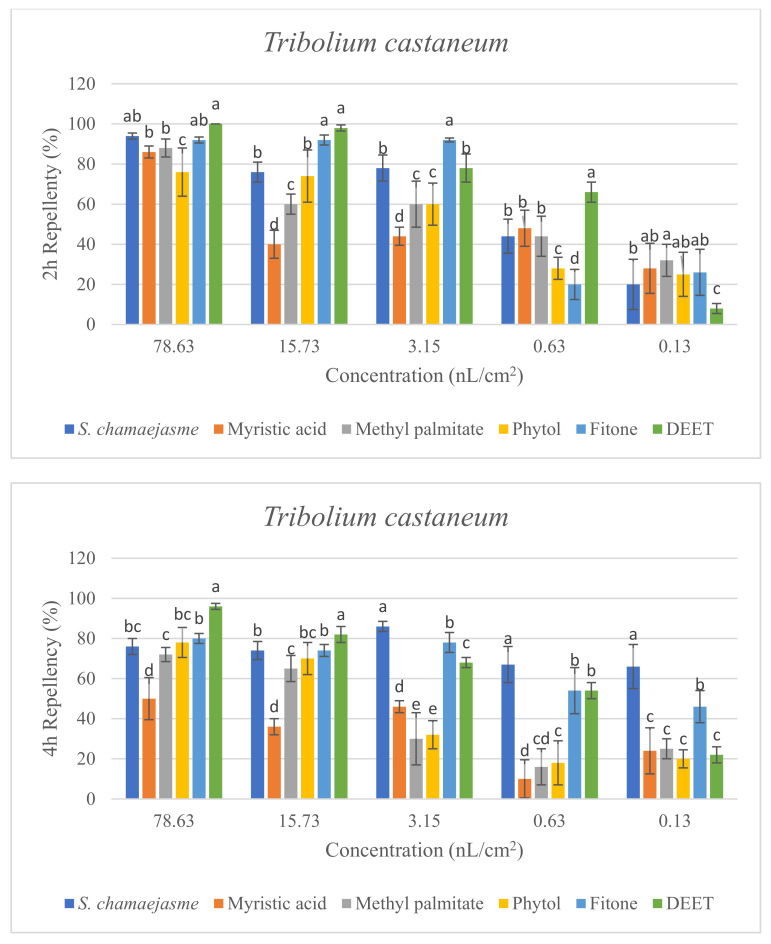

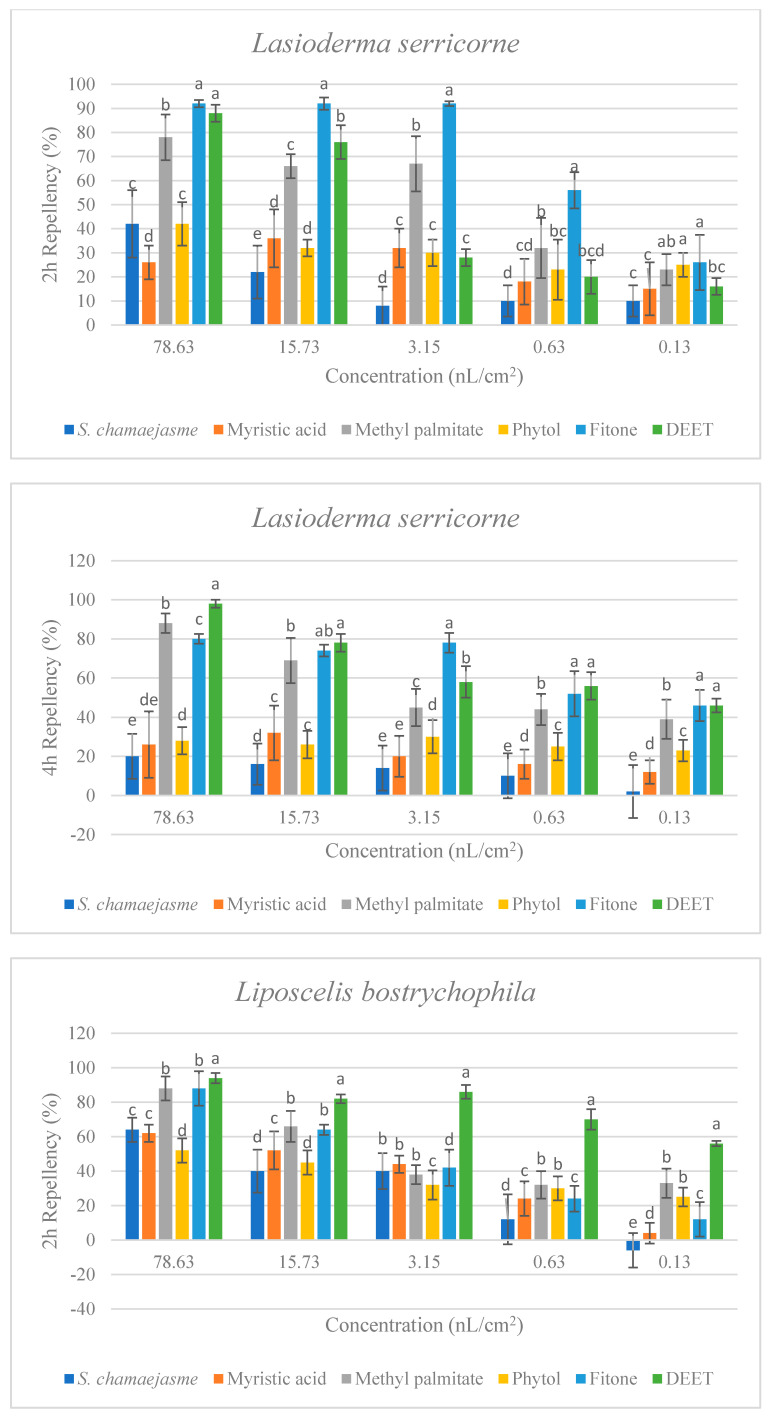

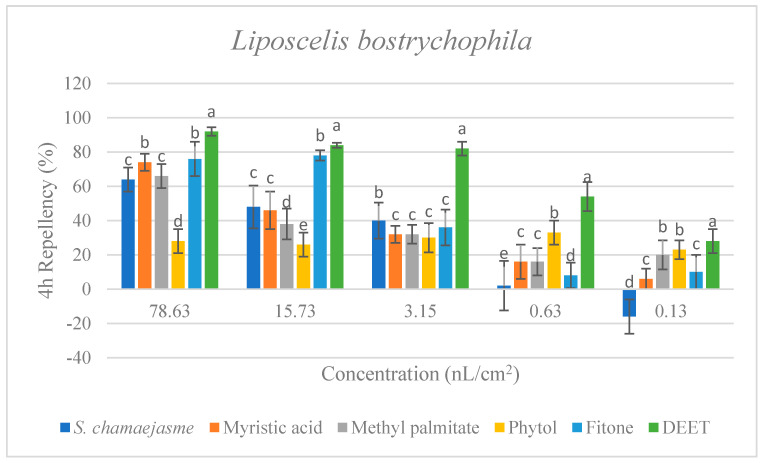
Percentage repellency (PR) of EO from *S. chamaejasme* and main monomeric compounds against *T. castaneum*, *L. serricorne*, and *L. bostrychophila* at 2 and 4 h after exposure. The meaning of the letters a, b, c, d, e in figures means in the same column followed by the same letters do not differ significantly in the tests of ANOVA and Tukey (*p* > 0.05).

In the repellent activity experiment against these three target insects, the repellent rate (%) at 2 h and 4 h was determined. The results are shown in Figure 4. The results of the repellent experiment showed that the EO from *S. chamaejasme* flowers had significant repellent effect on these three target insects at the different concentrations tested, and the repellent activity was reduced as the dose decreased. The EO exhibited higher repellency against the red flour beetle and the booklouse than the cigarette beetle after 2 and 4 h exposure. In addition, at the concentration of 3.15, 0.63 and 0.13 nL/cm^2^, the EO exhibited higher repellency against the red flour beetle at 4 h than that at 2 h, which indicated that EO could have a long-term effect on the red flour beetle. At the tested concentration of 3.15 nL/cm^2^, the EO showed higher repellency than the positive control of DEET against the red flour beetle after 2 and 4 h exposure. In addition, the results suggested the four chemical compounds in the EO from *S. chamaejasme* flowers played a certain role in repelling three target insects. Among them, myristic acid and phytol possessed higher repellency against the red flour beetle than against the cigarette and booklouse, whereas, methyl palmitate and fitone exhibited a significantly higher repellent effect than myristic acid and phytol against three insects, especially against the cigarette beetle. Additionally, at the concentration of 3.15 and 0.63 nL/cm^2^, the repellent effect of fitone and methyl palmitate was better than the positive control of DEET against the cigarette beetle after 2 h exposure, particularly fitone showed higher repellency than methyl palmitate against the cigarette beetle. To sum up, the volatile oil of *S. chamaejasme* flowers showed remarkable repellent activity against the red flour beetle, and also had a varying degree of repellent effect against the cigarette beetle and booklouse. The four monomeric compounds also have certain repellent activity against three insects, and the repellent effect of methyl palmitate and fitone were relatively higher than the other two compounds. In addition, the EO showed lower repellency than fitone against the cigarette beetle, which may be due to the synergistic or antagonistic effects of different components in the EO.

As we all know, the pests will gradually increase their own resistance when a single insecticide such as DEET has been used for a long time. Researchers have long been interested in the insecticidal activities of individual compounds, but gradually found that complete extracts were more active than their individual components [43,44]. Consequently, the complex mixture of secondary metabolites (mostly terpenoids) or plant extracts should be studied in future because they may provide multiple insecticidal mechanisms and ultimately reduce insect resistance [45,46]. In addition, the mixed EOs were made into microemulsions that still have the original insecticidal effect, which would improve the practicability of the mixtures of EOs [47]. Because the EO and some chemical compounds from *S. chamaejasme* flowers showed repellent bioactivity against stored product pests, the *S. chamaejasme* plant should be studied in future and considered for the preparation of a nanosized microemulsion, which may provide a good prospect for the control of storage insects.

## 3. Experiment

### 3.1. Plant Materials and Extraction

The raw materials were collected from 7 wild-growing populations of *chamaejasme*, identified in the region of Inner Mongolia (Table 7), China. The flowers of *S. chamaejasme* as experimental raw materials were used in this experiment. All medicinal herbs were identified as *Stellera chamaejasme* plants in *Stellera* genus of *Thymelaeaceae* family by the Plant Resources Appraisal Office of the Inner Mongolia Autonomous Region Food and Drug Inspection Institute. All samples had been identified as shown in Table 7. The fresh flowers of *S. chamaejasme* were selected and air-dried at room temperature. The flowers were subjected to hydrodistillation for a period of 6 h in a Clevenger (São Paulo, SP, Brazil) apparatus to extract the essential oil by using steam distillation equipment. The yellow-green EO was finally obtained and stored in an airtight container at 4 °C in refrigerator.

### 3.2. Experimental Insects

The three species of insects (*T. castaneum*, *L. serricorne*, and *L. bostrychophila*) used in the experiment were sampled from laboratory reproduction and verified by Professor Z. L. Liu (College of Plant Protection, China Agricultural University, Beijing, China). These three insect species were cultivated in a constant temperature and humidity chamber with temperature controlled at 30 ± 1 °C and humidity maintained at 70–80%. The booklouse was raised in conical bottles (50 mL) containing a mixture of milk powder, yeast, and flour (1:1:10, *w*/*w*). The red flour beetle and the cigarette beetle were bred in glass containers (0.5 L) with a proportional mixture of yeast and flour (1:10, *w*/*w*). All insects used in this experiment were 1–2 weeks old.

### 3.3. GC-FID and GC−MS Analysis

GC instrument (Agilent 7890A/5975C, Santa Clara, CA, USA) equipped with flame ionization detector (FID) was used in this experiment to establish the chromatographic fingerprints of 7 batches of EOs of *S. chamaejasme* flowers from different natural sites. The chromatographic column was HP–5MS (30 m × 0.32 mm × 0.25 μm). Nitrogen gas was used as the carrier gas at flow rate of 1.0 mL/min and the volume injection was 3.0 μL of 0.4% solution (diluted in *n*-hexane). The following measurement procedure was finally adopted after screening. The starting temperature of the column was set at 80 °C for 4 min, firstly rising to 160 °C at a speed of 10 °C/min, then rising to 184 °C at a speed of 2 °C/min, rising to 186 °C at a speed of 0.2 °C/min for 5 min, lastly rising to 280 °C at a speed of 5 °C/min for 25 min. The temperature of 280 °C was determined as the final detection temperature and 82.8 min was the total time of the chromatographic fingerprints.

In order to determine the chemical composition of the EO from *S. chamaejasme* flowers, a GC instrument (Agilent 6890N, Wilmington, DE, USA) combined with a mass spectrometer (Bruker 320, Folsom, CA, USA) was used in this experiment. The capillary column was DB–5MS (30 m × 0.25 mm × 0.25 μm) and the standard mass spectra was NIST 05 (Standard Reference Data, Gaithersburg, MD, USA). The starting temperature was set as 50 °C for 5 min, then rising to 290 °C at the speed of 10 °C/min for 11 min. Forty minutes chromatography in total was recorded and the volume injected was 1 μL of 1% solution (diluted in *n*-hexane). Each of the EO’s chemical constituents’ relative percentages was calculated by averaging peak area reports.

### 3.4. Experimental Data Analysis Methods

The chemical composition of EO from *S. chamaejasme* flowers was determined by GC−MS, identified by computer mass spectrometry (NIST 05) standard spectrometry database, and checked by reference to relevant literatures and according to the law of mass spectrometry lysis. Chromatographic fingerprint data were valued by the ‘Similarity Valuation system of TCM chromatographic fingerprints’ (China National Pharmacopoeia Commission) to analyze the similarity of *S. chamaejasme* flowers in different natural sites. The common characteristic peaks of the chromatographic fingerprints of samples from different areas could be confirmed by this system.

### 3.5. Repellent Test

The four compounds, fitone, methyl palmitate, myristic acid and phytol, are the main components in the EO of *S. chamaejasme* flowers, accounting for 52.764% of the total EO. Except phytol, the other three compounds are all terpenoids, a group of natural products usually possessing potential insecticidal activities. The repellent tests of EO from *S. chamaejasme* flowers and the 4 compounds (fitone, methyl palmitate, myristic acid, and phytol) against red flour beetle and cigarette beetle were carried out in 9 cm diameter petri dishes, while the repellent tests for the booklouse were implemented in 5.5 cm diameter petri dishes [48]. The EO and chemical compounds of *S. chamaejasme* flowers were dissolved in *n*-hexane to make a series of concentrations (78.63, 15.73, 3.15, 0.63, and 0.13 nL/cm^2^) to test the repellent rate of the red flour beetle and the cigarette beetle [49,50,51]. The specific experimental steps were to cut the filter paper with 9 cm diameter into two pieces on average. One half of paper was given 500 μL of *n*-hexane solvent as a blank control and the other half was treated with testing medicine in same volume. Both pieces of paper were stuck to the bottom of the petri dish with a glue stick after drying in the air for 30 s. Twenty insects were placed in the center of petri dish and covered. As for the booklouse, the EO and compounds were dissolved in *n*-hexane to prepare five different concentrations (63.17, 12.63, 2.53, 0.51, and 0.10 nL/cm^2^) to test the repellent rate of the booklouse [52,53]. The specific methods were the same as above, but the difference was that the dose was 150 μL. The testing insects were kept in a constant temperature and humidity box with temperature of 29 °C ± 1 °C and relative humidity of 70−80% [54,55]. At 2 and 4 h, the number of insects present on both sides of paper in each petri dish was recorded. Each concentration test was repeated five times. Meanwhile, the commercial insect repellent *N*, *N*-diethyl-3-methylbenzamide (DEET) was used as positive control [56,57]. The formula for calculating the percent repellency (PR) was as follows:

(1) $$PR\left(\%\right)=\left[\frac{\left(Nc-Nt\right)}{\left(Nc+Nt\right)}\right]\times 100$$

*Nc* represented the number of insects in the control groups, and *Nt* represented the number of insects in the testing group. The average repellent rate and standard deviation values were evaluated using Tukey’s test and variance analysis (ANOVA) by SPSS 26.0 software (IBM SPSS, Armonk, NY, USA).

## 4. Conclusions

In this research, the GC fingerprints of seven samples of EOs from *S. chamaejasme* flowers of different regions were established. Twenty-five common peaks were confirmed by the similarity evaluation system of Chinese medicine fingerprints and the similarity of the seven samples was between 0.9–1.0, indicating that the qualities of the seven samples of *S. chamaejasme* from various natural sites in the Inner Mongolia autonomous region were basically the same. In addition, the study evaluated the repellent activity of EO from *S. chamaejasme* flowers and its four chemical compounds against three common stored product insects for the first time. The experimental results showed that the volatile oil and its four chemical compounds from *S. chamaejasme* flowers had varying degrees of repellent effects against three target pests, particularly against the red flour beetle. The above results provided a basis for the development of plant-derived insecticides from *S. chamaejasme*, and also showed a good prospect for the prevention and control of stored product pests. However, the insecticidal mechanism of action of these active chemical components and the EO have not been clarified completely. The mutual synergistic or antagonistic relationships between the individual chemical compounds remain to be discovered by future research.

## Figures and Tables

**Table 1:** Chemical composition of essential oil from *S. chamaejasme* flowers.

Peak	Compounds	Molecular	Molecular	Relative	CAS Register	RI	Identification
No.		Formular	Weight	Content (%)	Number		Methods
1	Meso-2,5-Dimethyl-3,4-hexanediol	C_8_H_18_O_2_	146	6.846	22607–11–0	832	IR
2	Tetradeconic acid	C_14_H_28_O_2_	136	4.944	544–63–8	1021	IR
3	Ethanone,1-(2-methylcyclopropyl)-	C_6_H_10_O	98	16.151	930–56–3	868	IR
5	cis-Linalool oxide	C_10_H_18_O_2_	170	1.63	5989–33–3	1088	IR
6	Linalool	C_10_H_18_O	154	1.727	78–70–6	1106	IR
7	Terpineol	C_10_H_18_O	154	0.975	8006–39–1	1109	IR
8	4-(2,6,6-Trimethyl-1-cycloh exenyl)-3-buten-2-one	C_13_H_20_O	192	0.977	14901–07–6	1489	IR
9	Hexahydrofarnesyl acetone	C_18_H_36_O	268	38.978	502–69–2	1847	IR
10	Farnesyl acetone	C_18_H_30_O	262	2.463	1117–52–8	1915	IR
11	Methyl isohexadecanoate	C_17_H_34_O_2_	270	2.968	5129–60–2	1884	IR
12	Isophytol	C_20_H_40_O	296	3.293	505–32–8	1939	IR
13	trans-13-Octadecenoic acid	C_18_H_34_O_2_	282	1.013	693–71–0	1952	IR
14	n-Hexadecanoic acid	C_16_H_32_O_2_	256	4.854	1957–10–3	1958	IR
15	Phytol	C_20_H_40_O	296	3.988	150–86–7	2116	IR
16	Hentriacontane	C_31_H_64_	436	5.87	630–04–6	472	IR
	Total			96.677			

**Table 2:** Relative retention time of common peaks in seven batches of *S. chamaejasme* flowers chromatograms.

Batch Number	Peak Number												
	1(S)	2	3	4	5	6	7	8	9	10	11	12	13
1	1	1.118	1.1286	1.147	1.1871	1.2681	1.3128	1.343	1.713	1.7551	1.823	1.845	2.193
2	1	1.1184	1.1279	1.1479	1.1873	1.2678	1.3122	1.3344	1.7135	1.754	1.8231	1.8452	2.1926
3	1	1.118	1.1279	1.1484	1.1872	1.263	1.3113	1.3431	1.7139	1.7539	1.8199	1.8453	2.1928
4	1	1.1181	1.127	1.1473	1.1879	1.2677	1.3116	1.343	1.713	1.7535	1.8298	1.8399	2.1922
5	1	1.1188	1.1281	1.1477	1.1878	1.2733	1.3119	1.3344	1.7135	1.7529	1.823	1.8411	2.193
6	1	1.1186	1.1285	1.1475	1.1876	1.273	1.3115	1.3338	1.7132	1.7528	1.8231	1.84	2.1931
7	1	1.1184	1.1281	1.1477	1.1875	1.2686	1.3118	1.338	1.7134	1.7536	1.8236	1.8424	2.1928
**Mean value**	1	1.1184	1.1281	1.1477	1.1875	1.2686	1.3118	1.338	1.7134	1.7536	1.8236	1.8424	2.1928
**SD**	0	0.0003	0.0006	0.0004	0.0003	0.004	0.0006	0.005	0.0003	0.0008	0.003	0.003	0.0003
**Batch Number**	**Peak Number**												
	**1(S)**	**14**	**15**	**16**	**17**	**18**	**19**	**20**	**21**	**22**	**23**	**24**	**25**
1	1	2.2721	2.3225	2.3911	2.4281	2.519	2.5479	2.5715	2.601	2.6611	2.66	2.7461	2.861
2	1	2.2701	2.3221	2.3928	2.4283	2.5197	2.5471	2.571	2.6013	2.659	2.659	2.7465	2.8631
3	1	2.2711	2.3226	2.3923	2.4279	2.5191	2.5468	2.5711	2.6012	2.6592	2.6591	2.7451	2.8632
4	1	2.2712	2.3223	2.391	2.43	2.519	2.547	2.5712	2.6015	2.6591	2.6595	2.7422	2.8635
5	1	2.2713	2.3223	2.3915	2.4329	2.5192	2.5478	2.57	2.6017	2.6611	2.66	2.7459	2.8613
6	1	2.2715	2.3225	2.3917	2.4229	2.519	2.5477	2.5712	2.6013	2.6622	2.6601	2.7461	2.8616
7	1	2.271	2.3225	2.3911	2.4221	2.5292	2.5476	2.5711	2.6021	2.6625	2.6611	2.7499	2.8617
**Mena value**	1	2.2712	2.3224	2.3916	2.4275	2.5206	2.5474	2.571	2.6014	2.6606	2.6598	2.746	2.8622
**SD**	0	0.0006	0.0002	0.0007	0.004	0.004	0.0004	0.0005	0.0004	0.001	0.0007	0.02	0.001

**Table 3:** Relative peak area of common peaks in seven batches of *S. chamaejasme* flowers chromatograms.

Batch Number	Peak Number												
	1(S)	2	3	4	5	6	7	8	9	10	11	12	13
1	1	0.3763	0.4773	0.6765	0.2875	0.4223	0.3547	12.1436	0.2835	1.0318	0.8089	2.0941	0.516
2	1	0.6841	0.8574	0.5751	0.4675	0.8535	0.3583	12.5561	0.5778	1.065	0.8536	2.3984	0.6543
3	1	0.3744	0.4786	0.6643	0.2768	0.4931	0.3544	12.1687	0.2929	1.0511	0.8732	2.4921	0.4965
4	1	0.3761	0.4543	0.6685	0.2382	0.4228	0.3528	12.1569	0.273	1.0521	0.8079	2.2915	0.5243
5	1	0.377	0.4577	0.6989	0.2371	0.4263	0.3549	12.1344	0.2723	1.0532	0.8758	2.1917	0.5229
6	1	0.3561	0.4581	0.6875	0.287	0.432	0.3777	12.1551	0.2771	1.0311	0.8473	2.2891	0.5671
7	1	1.4539	0.3974	0.6636	0.2465	0.5342	0.3569	12.1675	0.2476	1.0437	0.8573	2.1674	0.5498
**Mean value**	1	0.57	0.51	0.66	0.28	0.51	0.36	12.21	0.32	1.05	0.85	0.28	0.55
**SD**	0	0.406	0.156	0.04	0.083	0.157	0.009	0.152	0.116	0.012	0.028	0.028	52
**Batch Number**	**Peak Number**												
	**1(S)**	**14**	**15**	**16**	**17**	**18**	**19**	**20**	**21**	**22**	**23**	**24**	**25**
1	1	0.7241	0.0835	0.658	2.2651	0.5683	0.157	0.2164	1.2155	0.1357	2.1361	0.7734	0.3782
2	1	0.7567	0.0846	0.7168	2.6846	0.5657	0.1537	0.6157	1.6137	0.5462	2.132	0.9863	0.5685
3	1	0.7276	0.0866	0.7685	2.2574	0.6836	0.1563	0.2677	1.2978	0.1748	2.1778	0.7936	0.3971
4	1	0.7332	0.0832	0.7754	2.7986	0.5693	0.1579	0.2572	1.2258	0.1785	2.1352	0.8659	0.4682
5	1	0.7231	0.0835	0.774	2.6771	0.5462	0.1578	0.2145	1.2561	0.1781	2.1466	0.5832	0.7892
6	1	0.7431	0.0832	0.7732	2.5789	0.5142	0.1589	0.2172	1.2635	0.148	2.1941	1.6835	1.3689
7	1	0.8865	0.0765	0.9786	2.4987	0.6357	1.5376	0.2188	1.3026	0.2453	2.3467	0.9458	0.5768
**Mean value**	1	0.69	0.83	0.78	2.54	0.58	0.35	0.29	1.31	0.23	2.18	0.95	0.65
**SD**	0	0.176	0.031	0.099	0.21	0.057	0.522	0.147	0.138	0.143	0.077	0.35	0.348

**Table 4:** Evaluation results of fingerprint similarities of essential oil from seven batches of *S. chamaejasme* flowers.

Sample Batch Number	S1	S2	S3	S4	S5	S6	S7	Control Fingerprint
**S1**	1	0.982	0.973	0.992	0.972	0.978	0.992	0.989
**S2**	0.982	1	0.994	0.988	0.981	0.992	0.981	0.995
**S3**	0.973	0.994	1	0.975	0.994	0.994	0.973	0.992
**S4**	0.992	0.988	0.975	1	0.98	0.98	0.988	0.991
**S5**	0.972	0.981	0.984	0.978	0.996	0.996	0.985	0.993
**S6**	0.978	0.992	0.994	0.98	1	1	0.987	0.997
**S7**	0.992	0.981	0.973	0.988	0.987	0.987	1	0.993
**Control fingerprint**	0.989	0.995	0.992	0.991	0.997	0.997	0.993	1

**Table 5:** Percentage of common peaks and noncommon peaks of the seven batches of *S. chamaejasme* flowers samples to the total peak area.

Sample Number	Percentage of Common Peaks to the Total Peak Area (%)	Percentage of Noncommon Peaks to the Total Peak Area (%)
1	97.08	2.92
2	96.62	3.38
3	97.70	2.30
4	97.71	2.29
5	98.16	1.84
6	97.37	2.63
7	97.68	2.32

**Table 6:** Test samples for experiment.

Test Samples	The Manufacturer	Serial Number
EO of *S. chamaejasme* flowers		
Myristic acid	Chengdu Refines Biotechnology Co., Ltd.	RFS-RO3911812016
Methyl palmitate	Chengdu Refines Biotechnology Co., Ltd.	RFS-Z06011812016
Phytol	Shanghai Yuanye Technology Co., Ltd.	S10B10Y85321
Fitone	Shanghai Yuanye Technology Co., Ltd.	S10A10Y85328

**Table 7:** Collected information of *S. chamaejasme* flowers.

Sample Numbers	Sample Resource	Altitude (m)	Sample Mass (kg)	Growth Environment
1	Wenduer Village, Xizhelim Town, Keyouzhong Banner, Inner Mongolia	863	2.02	Dry sunny hillside
2	Zhabente Banner, Xing’an League, Inner Mongolia	300	2.54	Grassland
3	Sanggendalai, Zhenglan Banner, Xilinhot City, Xilin Gol League, Inner Mongolia	635	2.27	River beach terrace
4	Alahada Village, Zalut Banner, Tongliao City, Inner Mongolia	412	2.46	Grassland
5	Bayanbaoligao, Tianshan Town, Aluhorqin Banner, Chifeng City, Inner Mongolia	510	2.34	Hillside
6	Taohemusumu in keshiqian banner, Xing’an League, Inner Mongolia		2.69	Semi-cultivation
7	Daban Town, Balin Right Banner, Chifeng City, Inner Mongolia	316	2.43	Half slope

## Data Availability

All research data has been presented in this article and available from authors.

## Sample Availability

Samples of the compounds are available from the authors.
